# Causal associations between site-specific cancer and diabetes risk: A two-sample Mendelian randomization study

**DOI:** 10.3389/fendo.2023.1110523

**Published:** 2023-02-13

**Authors:** Rong Xu, Tingjin Zheng, Chaoqun Ouyang, Xiaoming Ding, Chenjin Ge

**Affiliations:** ^1^ Department of Pharmacy, Quanzhou Medical College, Quanzhou, China; ^2^ Department of Clinical Laboratory, Quanzhou First Hospital Affiliated to Fujian Medical University, Quanzhou, China; ^3^ Department of Basic Medicine, Quanzhou Medical College, Quanzhou, China; ^4^ Department of Medical Imaging, Shanghai Hospital of Traditional Chinese Medicine, Shanghai University of Traditional Chinese Medicine, Shanghai, China

**Keywords:** site-specific cancer, diabetes, lymphoid leukaemia, Mendelian randomization, causality

## Abstract

**Background:**

Both cancer and diabetes are complex chronic diseases that have high economic costs for society. The co-occurrence of these two diseases in people is already well known. The causal effects of diabetes on the development of several malignancies have been established, but the reverse causation of these two diseases (e.g., what type of cancer can cause T2D) has been less investigated.

**Methods:**

Multiple Mendelian randomization (MR) methods, such as the inverse-variance weighted (IVW) method, weighted median method, MR-Egger, and MR pleiotropy residual sum and outlier test, were performed to evaluate the causal association of overall and eight site-specific cancers with diabetes risk using genome-wide association study summary data from different consortia, such as Finngen and UK biobank.

**Results:**

A suggestive level of evidence was observed for the causal association between lymphoid leukaemia and diabetes by using the IVW method in MR analyses (*P* = 0.033), indicating that lymphoid leukaemia increased diabetes risk with an odds ratio of 1.008 (95% confidence interval, 1.001-1.014). Sensitivity analyses using MR-Egger and weighted median methods showed consistent direction of the association compared with the IVW method. Overall and seven other site-specific cancers under investigation (i.e., multiple myeloma, non-Hodgkin lymphoma, and cancer of bladder, brain, stomach, lung, and pancreas) were not causally associated with diabetes risk.

**Conclusions:**

The causal relationship between lymphoid leukaemia and diabetes risk points to the necessity of diabetes prevention amongst leukaemia survivors as a strategy for ameliorating the associated disease burden.

## Introduction

One of the twenty-first century’s major threats to public health is the elevation of diabetes mellitus prevalence worldwide (1). An initial stage of insulin resistance and compensatory hyperinsulinemia which contribute to β-cell failure defines type 2 diabetes (T2D) (2). T2D is characterized by chronic hyperglycaemia, which damages end organs over time (2). The World Health Organization reports that out of six deaths, one is attributed to cancer, which makes cancer the second primary cause of mortality worldwide (3). Both cancer and diabetes are complex chronic diseases and have high economic costs for society. The co-occurrence of these two diseases in people has already been reported for more than 50 years (4). It is presumed that these two diseases may have similar developmental pathways, such as the malfunction of immunological regulation and cytokine activity (5). Common risk factors, such as obesity, genetic predisposition, and exposure to certain environmental factors, have been identified in the development of cancer and diabetes (5, 6). Given that abdominal adiposity has been found to promote a proinflammatory condition throughout the body, which increases the risk of cancer and diabetes, obesity has been proposed as one of the underlying reasons for these two diseases (6).

Epidemiological evidence has indicated that several malignancies are more likely to occur in people with T2D (7). For instance, diabetes significantly increases the relative risk of liver and pancreatic cancer (PC) (8, 9), but less evidence has been observed for other cancers. Because the development of some malignancies can precede and cause T2D, the potential reverse causation of these two diseases should also be considered. For instance, PC is likely to promote the development of T2D (10). According to a recent study from Korea, cancer can enhance the risk of developing diabetes among cancer survivors, independent of conventional diabetes risk factors (11). The diabetes risk was most significant in the first two years after cancer diagnosis, and elevated risk was continuously observed for as long as 10 years (11). Moreover, circulating cytokines aggravate hyperglycaemia in cancer patients by promoting insulin resistance and increasing hepatic gluconeogenesis (12). A standard tumour marker for PC is a higher level of CA19-9, and elevated serum CA19-9 levels have been related to the severity of inadequate glucose regulation (13, 14). It has been proposed that survivors of cancer treatment are at higher risk for endocrinopathies, such as diabetes and metabolic syndrome, for the rest of their lives (15). For example, recent work has revealed that diabetes is more likely to develop in people who survived childhood cancer (15). In addition, a long latency may exist between cancer treatment and the onset of different treatment-related conditions, emphasizing the necessity for lifelong awareness and monitoring (16).

Observational epidemiological research can be hampered by various potential biases caused by residual confounding (17). Moreover, the possible reverse causation of the exposure and outcome in these works makes it difficult to determine the direction of the correlations (17). The Mendelian randomization (MR) method, which uses genetic variants as instrumental variables, can infer the causal effects of exposure on outcomes. Because genetic variations are fixed at birth and normally cannot be modified by outcomes, MR analyses are less affected by reverse causality (18). Considering that the effects of cancer from different sites on diabetes risk may be different (19), the current study used the MR method to estimate the causal effects of overall and eight site-specific cancers on the risk of diabetes.

## Methods

### Study design

MR examines the causal relationship between exposures and diseases using genetic variants (e.g., single nucleotide polymorphisms [SNPs]) as instrumental variables (IVs). In our analyses, the summary statistics of IVs were taken from genome-wide association study (GWAS) datasets of overall and site-specific cancers. Three requirements should be met for the selection of IVs. First, IVs are not directly associated with outcomes, and they only influence outcomes through exposure. Second, strong correlations exist between IVs and exposure. Third, IVs are not associated with the confounders (no horizontal pleiotropy exists). An MR framework was employed using GWAS summary data from different consortia to evaluate the causal association between overall and eight site-specific cancers and diabetes risk.

### Data sources

Summary-level genetic data for overall and site-specific cancers were gathered from Finngen (20), the international lung cancer consortium (ILCCO) (21), the UK biobank (UKB) (22) and the genetic epidemiology research on aging (GERA) (23). **Supplementary Table 1** provides more information on the data sources. GWAS datasets were used to extract the IVs for overall and lung cancer, in which the SNPs reached a genome-wide significance level (*P* < 5 × 10^–8^). We lowered the *P* value threshold for including SNPs as IVs to *P* < 1 × 10^-5^ if fewer than five IVs were selected (**Supplementary Table 1**). This threshold-lowering method has been previously adopted in MR studies (24). SNPs within 10,000 kb of each other were then clumped, with a linkage disequilibrium threshold of R2 > 0.001. The F-statistics of the IVs, an indicator of the ability of the IVs to predict the exposures (25), were estimated, and all exposures had F-statistics higher than 10 (**Supplementary Table 2**). The GWAS datasets for T2D, as the outcome, were from the Diabetes Meta-analysis of Trans-ethnic Association Studies (DIAMANTE) consortium (26).

### Statistical analysis

The major method used to ascertain the relationships between different types of cancer and diabetes risk was the inverse-variance weighted (IVW) MR method. For sensitivity analyses, the weighted median (WM) method, MR-Egger, and MR pleiotropy residual sum and outlier (MR-PRESSO) test were also conducted. The potential heterogeneity was estimated by Cochrane’s Q statistic, and the potential pleiotropy was assessed by the intercept of the MR-Egger test. Scatter plots were used to present the results of different MR methods. The estimate of the effect of SNPs after removing each SNP one by one was achieved by “leave-one-out” analysis. The causal effects of overall and site-specific cancer were represented using odds ratios (ORs) and 95% confidence intervals (CIs). The statistical significance of the MR analyses was adjusted using Bonferroni correction. The testing results that did not survive Bonferroni correction but had a *P* < 0.05 were defined as associations with suggestive level of evidence. R software was used for these analyses, in which the “TwoSampleMR” and “MR-PRESSO” R packages were employed.

## Results

**Figure 1:**
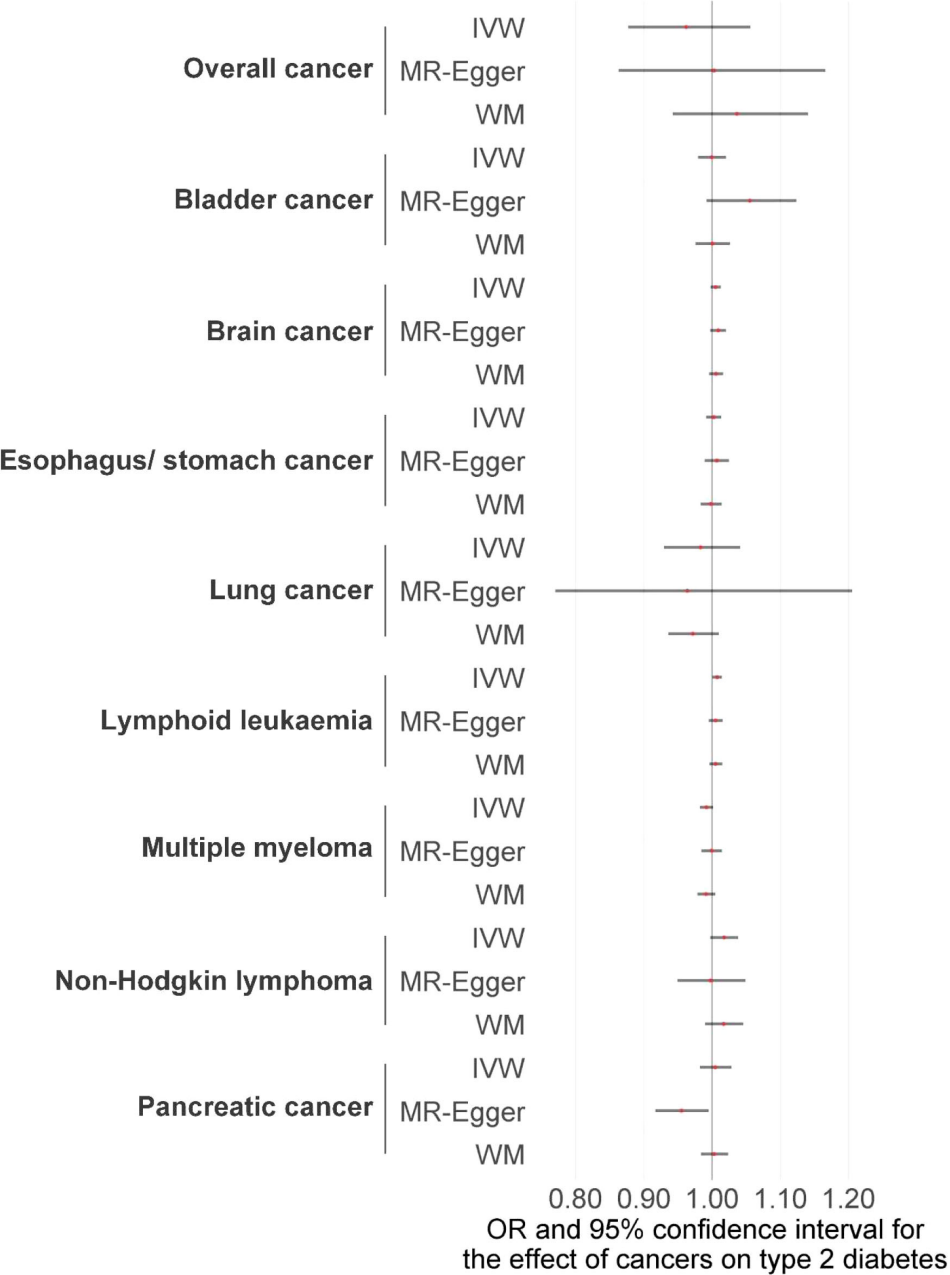
The potential causal relationships between site-specific cancer and diabetes risk were examined using various MR methods, including IVW, MR-Egger, and WM. IVW, inverse-variance weighted method; MR, Mendelian randomization; WM, weighted median method; OR, odds ratio.

**Figure 2:**
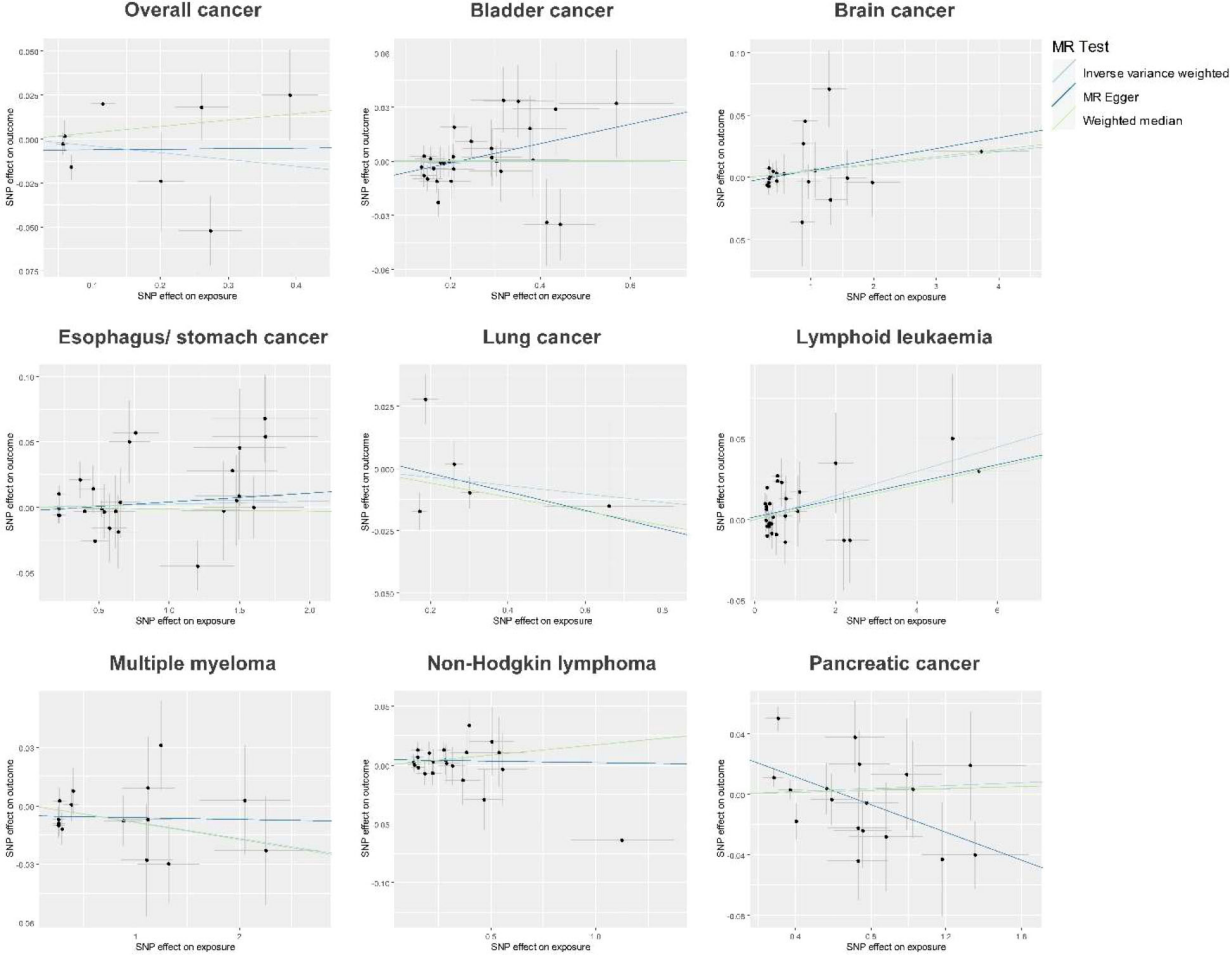
Scatter plots of the MR analyses showing the potential causal associations of site-specific cancer with diabetes. MR, Mendelian randomization; SNP, single nucleotide polymorphism.

**Figure 3:**
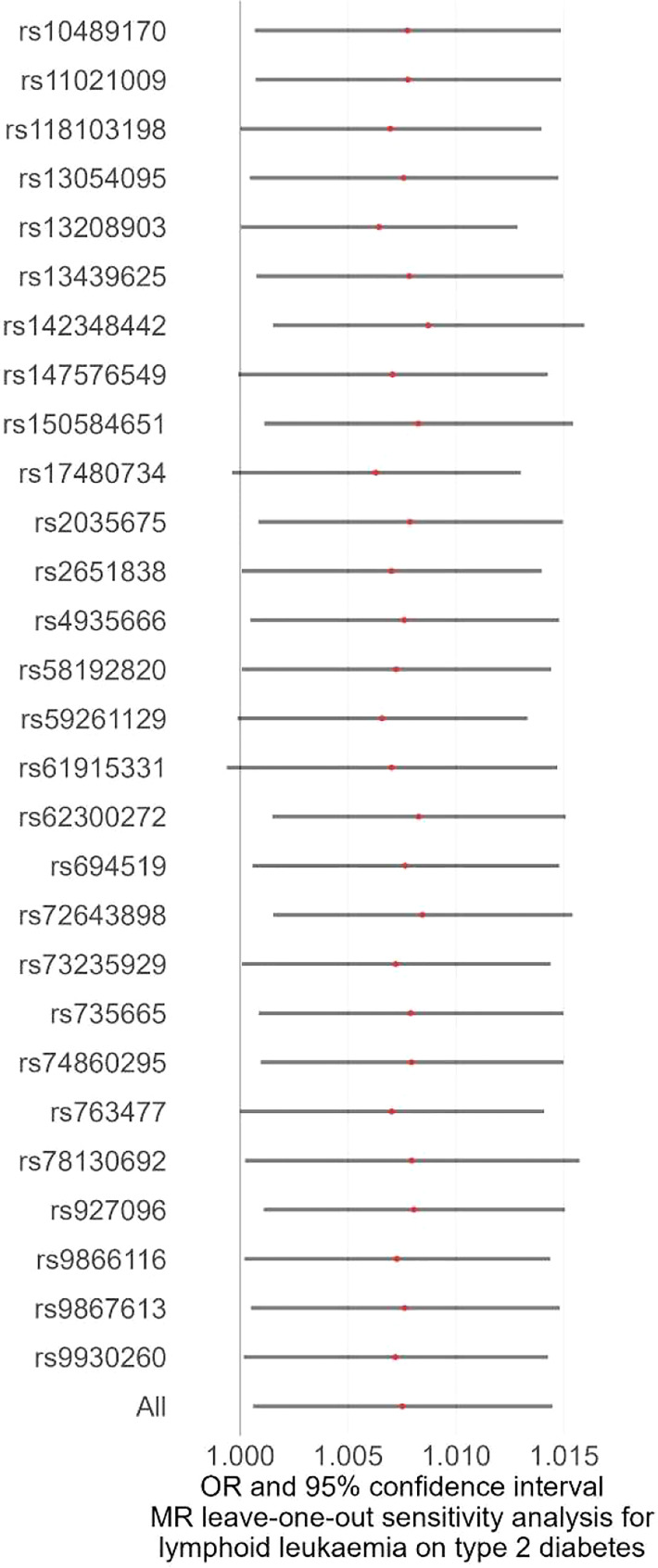
Leave-one-out analysis as a sensitivity analysis to examine the causal association between lymphoid leukaemia and diabetes. MR, Mendelian randomization; OR, odds ratio.

We first performed the MR analyses to examine the possible causal association of overall and eight site-specific cancers with diabetes using GWAS summary statistics from various consortia. Detailed information, as well as *P* threshold for IV selection for each GWAS summary dataset, is given in **Supplementary Table 1**. The results indicated that none of the tested associations survived Bonferroni correction with a *P* threshold of 0.05/9 = 0.006, but a suggestive level of evidence was observed for the causal association between lymphoid leukaemia and diabetes (IVW method, *P* = 0.033), indicating that lymphoid leukaemia increased diabetes risk, with an OR of 1.008 (95% CI, 1.001-1.014) (**Figures 1**, **2**; **Supplementary Figure 1**, **Supplementary Table 3**). The F-statistic of the IVs used in these analyses ranged from 15.7 to 151.5, with a mean of 25.4, suggesting strong ability of the IVs to predict the exposures (**Supplementary Table 2**). For the observed causal association between lymphoid leukaemia and diabetes, sensitivity analyses using the MR-Egger and WM methods showed a consistent direction of the association compared with the IVW method. In addition, the leave-one-out sensitivity analysis revealed that the association of lymphoid leukaemia with diabetes became marginally significant after removing several SNPs, including rs147576549, rs17480734, rs59261129, rs61915331, and rs763477, with a *P* value ranging from 0.050 to 0.072 (**Figure 3**). Furthermore, no significant heterogeneity or horizontal pleiotropy was detected in the analysis of causality between lymphoid leukaemia and diabetes (**Supplementary Tables 4**, **5**, respectively). MR-PRESSO consistently revealed no outlier IV in the analysis of lymphoid leukaemia, and the results were identical for the analyses of bladder cancer and PC after correcting for the identified outlier SNPs (**Supplementary Table 6**).

## Discussion

Our study screened the possible causal association of a total of eight site-specific cancers with diabetes using MR methods based on GWAS summary datasets, and we found that lymphoid leukaemia was causally associated with diabetes risk. This observation is also reflected by the results of MR-Egger and WM MR analyses that showed a consistent direction of association. In addition, the MR-Egger intercept test and MR-PRESSO global test revealed that the causal association between lymphoid leukaemia and diabetes was not due to horizontal pleiotropy.

A class of deadly hematologic malignancies known as leukaemia is defined by malignant growth of white blood cells and their precursor cell (27). On the one hand, an increased leukaemia risk has been reported in patients with diabetes. For instance, a study in Sweden showed that patients with T2D had a noticeably higher incidence of leukaemia after hospitalization (28). Meta-analysis of 11 publications indicated that the OR of leukaemia for people with T2D was estimated to be 1.22 (29). On the other hand, leukaemia has been proposed as one of the childhood cancers that leads to higher risk of diabetes (30). Indeed, childhood cancer survivors were more likely to develop diabetes compared with their sibling controls according to one study from the childhood cancer survivor study (CCSS) group (31). Consistent results were observed in studies conducted in Scandinavia (32) and Canada (33).

Several mechanisms underlying the higher diabetes risk in patients with leukaemia have been proposed. Leukaemia cells can directly infiltrate the pancreas (34), and chemotherapeutic treatment using L-asparagine can also lead to β-cell malfunction, causing hyperglycaemia in acute lymphocytic leukaemia (34), one of the most prevalent cancers among children (35). For chronic lymphocytic leukaemia, one case report indicated that a patient developed diabetes after being treated with fludarabine and cyclophosphamide therapy, which could potentially disrupt the local immune-regulatory balance (36). Corticosteroids are normally used as an integral part of combination chemotherapy in leukaemia treatment (37). However, some complications might arise during the usage of corticosteroids, of which two of the most common are hyperglycaemia and chemotherapy-induced diabetes (CID) (38). The development of diabetes after abdominal radiation is often linked to damage to the pancreas tail induced by the radiation, which leads to pancreatic insufficiency (39). For hematopoietic cell transplantation patients suffering from high-risk hematologic cancers, the precondition is normally achieved by total body irradiation (TBI) (40). The entire body is exposed to radiation during TBI, which affects the hypothalamic-pituitary axis and increases the risk of endocrinopathies (e.g., growth hormone deficiency) in cancer survivors (41). The risks of developing diabetes have been documented amongst children survivors exposed to TBI treatment, with a 12.6-fold risk ratio compared with their sibling controls (31). The major pathophysiologic mechanisms that contribute to the post-TBI development of diabetes have been proposed to be insulin resistance and hyperinsulinemia, rather than pancreatic insufficiency (16). It is also not uncommon for survivors of TBI exposure to present abnormality processes, such as altered adipokines and occurrence of inflammation (42).

CID contributes to poor clinical outcomes in leukaemia patients (43), and the underlying reasons could be multifactorial. One explanation is the increased susceptibility to infections in patients with CID undergoing intensive chemotherapy (44). Hyperglycaemia and hyperinsulinemia can further stimulate the neoplastic process, leading to unfavourable clinical outcomes in patients with leukaemia and CID (45). In patients suffering from acute myeloid leukaemia, researchers also reported an alteration in the glucose metabolism signature, which contributes to undesirable clinical outcomes (46). Thus, early commencement of CID screenings and relevant strategies to reduce its negative impact is advised because cancer survivors have an elevated chance of developing premature cardiovascular morbidity (47). Further research is warranted to elucidate the complex metabolic abnormality in cancer survivors, which could guide preventive and therapeutic endeavours to improve the quality of life of cancer survivors.

The association between cancer and diabetes can be site specific. For example, the risks of developing diabetes have been reported to be comparatively higher for survivors of PC compared with other types of cancers (48). A significant portion of patients recently diagnosed with PC present hyperglycaemia or T2D (49). In addition, T2D is alleviated after tumour removal, which reinforces the idea that T2D is related to PC (50). The risk of diabetes is elevated by PC because it promotes the secretion of insulin that leads to insulin resistance (51). Furthermore, pancreatic tissue destruction with an accompanying β-cell loss can also occur in patients with PC, which contributes to the development of diabetes (52). However, the causal effects of PC on T2D subtypes may be different. One MR analysis suggested that PC is causally associated with newly onset T2D but not long-standing T2D (53). The GWAS summary dataset of T2D used in our MR analysis did not separate subtypes of T2D, and the results indicated no causal association between PC and T2D. Similar to PC, six other site-specific cancers under investigation, including multiple myeloma, non-Hodgkin lymphoma and cancers of the bladder, brain, stomach, and lung, were also not causally associated with diabetes.

There were several areas of strength in this study. First, we employed an MR design to reduce the biases that can be introduced by reverse causality and residual confounding in conventional observational studies, which may lead to false-positive results. Second, numerous SNPs were used as IVs for overall and site-specific cancers, which was essential in facilitating the analysis of horizontal pleiotropy. Third, for sensitivity analyses aimed at estimating pleiotropy, several MR methods, such as MR-PRESSO and MR-Egger, were utilized. Lastly, the participants within the initial GWAS were mainly of European descent, which helped to reduce the bias attributable to population stratification. Despite the strengths, there were also several shortcomings in the present study, a key of which was the inability to completely exclude the possible effect of pleiotropy. Additionally, the interpretation of the results was limited to a certain ethnicity because the GWAS summary datasets were of European origin.

## Conclusion

This comprehensive MR analysis has established a causal relationship between lymphoid leukaemia and diabetes risk, which points to the necessity of diabetes prevention amongst leukaemia survivors as a strategy for ameliorating the associated disease burden.

## Data availability statement

The original contributions presented in the study are included in the article/**Supplementary Material**. Further inquiries can be directed to the corresponding authors.

## Ethics statement

The GWAS used in the current work were approved by their relevant review board, and informed consent were collected from all participants.

## Author contributions

RX and CG concepted the study. RX, TZ, CO, and XD performed the statistical analyses, and drafted the manuscript. All authors contributed to the article and approved the submitted version. 

## Supplementary material

Supplementary Figure 1 Funnel plot of the MR analyses investigating the causal effects of site-specific cancer and diabetes. Abbreviations: MR: Mendelian randomization; SE: standard error; IVs: instrumental variables.

Supplementary Table 1 Information of GWAS summary datasets used in MR analyses.

Supplementary Table 2 The F-statistics of IVs.

Supplementary Table 3 MR analysis results.

Supplementary Table 4 Heterogeneity test results.

Supplementary Table 5 MR-Egger pleiotropy test results.

Supplementary Table 6 MR-PRESSO test results.
