# Supplementary material for: Causal associations between site-specific cancer and diabetes risk: A two-sample Mendelian randomization study
Source: Front Endocrinol (Lausanne). 2023 Feb 13;14:1110523. doi: 10.3389/fendo.2023.1110523 (PMC9968794; doi:10.3389/fendo.2023.1110523)
Supplement: Supplementary Figure 1 — Funnel plot of the MR analyses investigating the causal effects of site-specific cancer and diabetes. Abbreviations: MR: Mendelian randomization; SE: standard error; IVs: instrumental variables. [file DataSheet_1.docx]

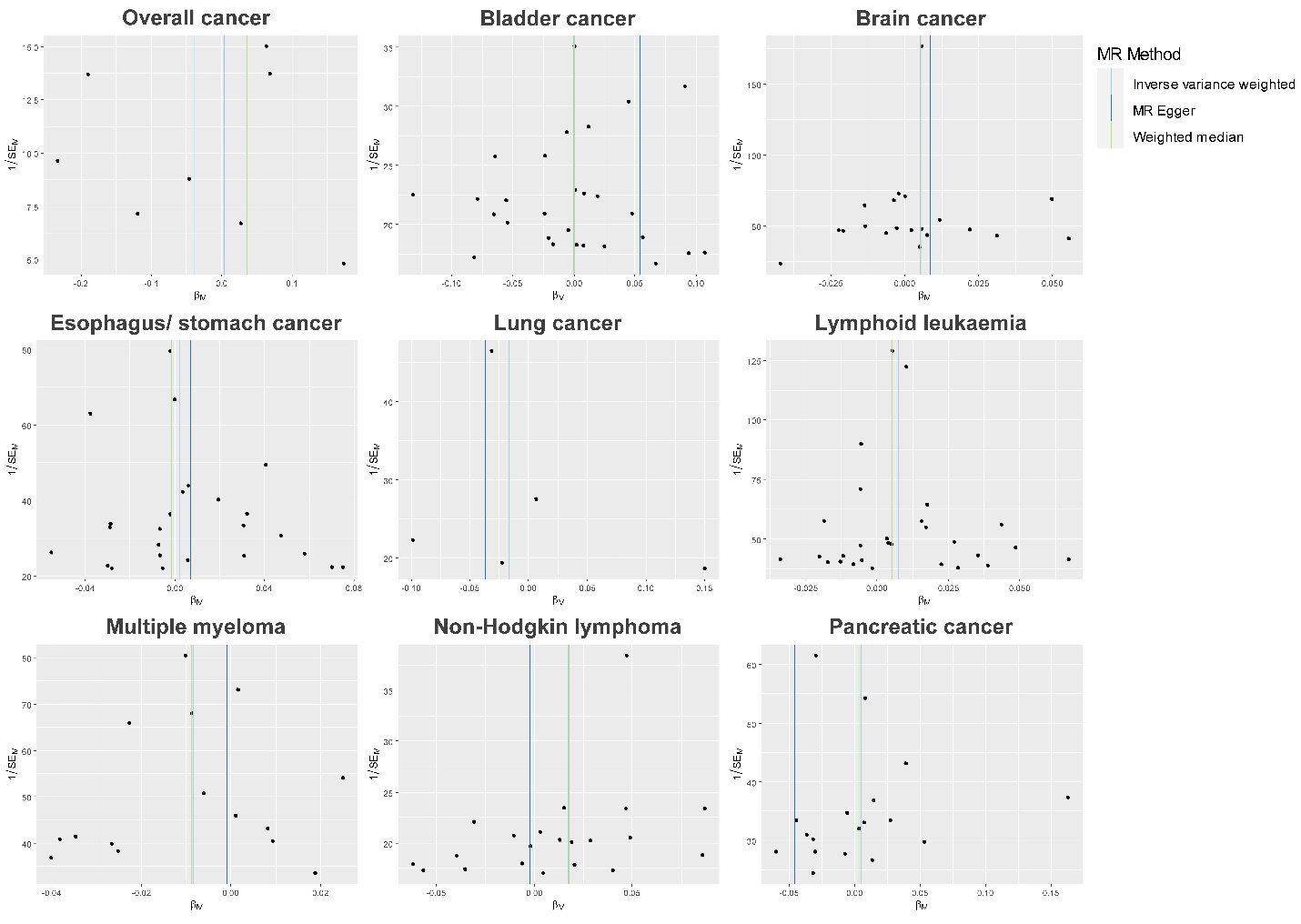


**Supplementary Figure 1**. Funnel plot of the MR analyses investigating the causal effects of site-specific cancer and diabetes. Abbreviations: MR: Mendelian randomization; SE: standard error; IVs: instrumental variables.

**Supplementary Table 1**. Information of GWAS summary datasets used in MR analyses.

| Traits | Consortium | Sample size | *P* threshold for IV selection |
| --- | --- | --- | --- |
| Overall cancer | Finngen | 218792 | 5.00E-08 |
| Bladder cancer | The UKB and GERA | 412592 | 5.00E-05 |
| Brain cancer | Finngen | 218792 | 5.00E-05 |
| Esophagus/ stomach cancer | The UKB and GERA | 411441 | 5.00E-05 |
| Lung cancer | ILCCO | 27209 | 5.00E-08 |
| Lymphoid leukaemia | Finngen | 218792 | 5.00E-05 |
| Multiple myeloma | Finngen | 218792 | 5.00E-05 |
| Non-Hodgkin lymphoma | The UKB and GERA | 412750 | 5.00E-05 |
| Pancreatic cancer | The UKB and GERA | 411013 | 5.00E-05 |
| Type 2 diabetes | DIAMANTE | 898130 | NA |
| Abbreviation: MR: Mendelian randomization; GWAS: genome-wide association studies; UKB: UK Biobank; GERA: Genetic Epidemiology Research on Aging; ILCCO: International Lung Cancer Consortium; DIAMANTE: Diabetes Meta-analysis of Trans-ethnic Association Studies; NA: not available; IV: Instrumental variable. | | | |

**Supplementary Table 2**. The F-statistics of IVs.

| Type of cancer | SNP | EA | OA | F-Statistic |  |
| --- | --- | --- | --- | --- | --- |
| Overall cancer | rs10111694 | A | T | 31.7 |  |
| Overall cancer | rs11200014 | A | G | 38.1 |  |
| Overall cancer | rs145922598 | T | C | 42.9 |  |
| Overall cancer | rs1800057 | G | C | 34.4 |  |
| Overall cancer | rs4268748 | C | T | 44.2 |  |
| Overall cancer | rs59957953 | T | A | 39.7 |  |
| Overall cancer | rs62235753 | T | C | 88.1 |  |
| Overall cancer | rs78378222 | G | T | 33.9 |  |
| Bladder cancer | rs10094872 | A | T | 43.9 |  |
| Bladder cancer | rs10492036 | T | C | 19.6 |  |
| Bladder cancer | rs10815684 | T | C | 21.0 |  |
| Bladder cancer | rs11055684 | T | G | 20.1 |  |
| Bladder cancer | rs112037107 | C | T | 21.0 |  |
| Bladder cancer | rs116718550 | G | A | 20.0 |  |
| Bladder cancer | rs11724531 | A | G | 46.9 |  |
| Bladder cancer | rs117903242 | C | T | 20.3 |  |
| Bladder cancer | rs12194317 | T | C | 20.2 |  |
| Bladder cancer | rs12641941 | G | T | 20.2 |  |
| Bladder cancer | rs12773005 | G | A | 22.9 |  |
| Bladder cancer | rs13203636 | G | C | 20.4 |  |
| Bladder cancer | rs13242427 | G | A | 20.1 |  |
| Bladder cancer | rs13261269 | C | T | 21.5 |  |
| Bladder cancer | rs16831845 | T | C | 19.7 |  |
| Bladder cancer | rs17793392 | A | G | 20.9 |  |
| Bladder cancer | rs251679 | T | C | 20.1 |  |
| Bladder cancer | rs2736103 | T | C | 26.1 |  |
| Bladder cancer | rs4075596 | T | C | 28.6 |  |
| Bladder cancer | rs4350702 | G | T | 20.6 |  |
| Bladder cancer | rs56297045 | G | A | 27.2 |  |
| Bladder cancer | rs7041648 | T | G | 19.7 |  |
| Bladder cancer | rs72882571 | G | A | 19.7 |  |
| Bladder cancer | rs75586170 | T | C | 19.9 |  |
| Bladder cancer | rs76088467 | A | G | 31.2 |  |
| Bladder cancer | rs76389793 | T | C | 20.5 |  |
| Bladder cancer | rs78586378 | C | T | 19.7 |  |
| Bladder cancer | rs814583 | T | C | 21.5 |  |
| Brain cancer | rs10059500 | T | C | 21.3 |  |
| Brain cancer | rs1021619 | A | G | 23.6 |  |
| Brain cancer | rs10966834 | G | A | 20.8 |  |
| Brain cancer | rs11207597 | C | G | 22.8 |  |
| Brain cancer | rs112102788 | A | C | 26.0 |  |
| Brain cancer | rs1194817 | T | C | 20.0 |  |
| Brain cancer | rs12804088 | C | T | 19.6 |  |
| Brain cancer | rs1378254 | T | G | 24.0 |  |
| Brain cancer | rs139696720 | T | C | 24.9 |  |
| Brain cancer | rs146130523 | C | T | 21.1 |  |
| Brain cancer | rs147958197 | C | T | 21.9 |  |
| Brain cancer | rs148088011 | A | T | 25.4 |  |
| Brain cancer | rs1565017 | A | G | 22.5 |  |
| Brain cancer | rs2285457 | G | A | 19.9 |  |
| Brain cancer | rs2964708 | C | T | 19.6 |  |
| Brain cancer | rs4658437 | G | A | 20.8 |  |
| Brain cancer | rs55737523 | G | C | 23.0 |  |
| Brain cancer | rs73161249 | G | C | 20.3 |  |
| Brain cancer | rs77671862 | A | C | 20.1 |  |
| Brain cancer | rs9542683 | C | A | 20.4 |  |
| Esophagus/ stomach cancer | rs1000441 | C | T | 23.2 |  |
| Esophagus/ stomach cancer | rs10029873 | A | G | 20.7 |  |
| Esophagus/ stomach cancer | rs10925740 | T | C | 23.9 |  |
| Esophagus/ stomach cancer | rs116267209 | G | A | 22.6 |  |
| Esophagus/ stomach cancer | rs117539707 | G | C | 20.5 |  |
| Esophagus/ stomach cancer | rs117661056 | G | A | 20.0 |  |
| Esophagus/ stomach cancer | rs11842832 | T | C | 22.2 |  |
| Esophagus/ stomach cancer | rs13391440 | T | A | 20.3 |  |
| Esophagus/ stomach cancer | rs141337710 | T | C | 20.7 |  |
| Esophagus/ stomach cancer | rs141603928 | T | C | 21.6 |  |
| Esophagus/ stomach cancer | rs143542784 | G | A | 19.7 |  |
| Esophagus/ stomach cancer | rs143621906 | G | A | 20.1 |  |
| Esophagus/ stomach cancer | rs147939405 | C | T | 19.9 |  |
| Esophagus/ stomach cancer | rs180759666 | C | T | 20.7 |  |
| Esophagus/ stomach cancer | rs1996131 | T | C | 21.4 |  |
| Esophagus/ stomach cancer | rs2209102 | G | A | 21.3 |  |
| Esophagus/ stomach cancer | rs34240847 | A | G | 21.8 |  |
| Esophagus/ stomach cancer | rs3778354 | G | T | 19.7 |  |
| Esophagus/ stomach cancer | rs41313954 | C | T | 20.1 |  |
| Esophagus/ stomach cancer | rs58714294 | G | C | 20.2 |  |
| Esophagus/ stomach cancer | rs618688 | G | A | 25.0 |  |
| Esophagus/ stomach cancer | rs62134018 | G | A | 26.1 |  |
| Esophagus/ stomach cancer | rs71448917 | C | T | 22.3 |  |
| Esophagus/ stomach cancer | rs72745750 | C | T | 21.0 |  |
| Esophagus/ stomach cancer | rs7512851 | G | A | 25.3 |  |
| Lung cancer | rs11571818 | C | T | 15.7 |  |
| Lung cancer | rs37004 | T | C | 78.6 |  |
| Lung cancer | rs446975 | T | G | 133.3 |  |
| Lung cancer | rs501942 | T | C | 30.0 |  |
| Lung cancer | rs8040868 | C | T | 151.5 |  |
| Lymphoid leukaemia | rs10489170 | G | A | 21.0 |  |
| Lymphoid leukaemia | rs11021009 | T | C | 22.0 |  |
| Lymphoid leukaemia | rs118103198 | A | G | 20.8 |  |
| Lymphoid leukaemia | rs13054095 | T | A | 24.4 |  |
| Lymphoid leukaemia | rs13208903 | A | G | 20.1 |  |
| Lymphoid leukaemia | rs13439625 | G | A | 20.9 |  |
| Lymphoid leukaemia | rs142348442 | T | G | 23.0 |  |
| Lymphoid leukaemia | rs147576549 | T | C | 21.2 |  |
| Lymphoid leukaemia | rs150584651 | C | T | 23.6 |  |
| Lymphoid leukaemia | rs17480734 | C | A | 25.5 |  |
| Lymphoid leukaemia | rs2035675 | G | A | 22.4 |  |
| Lymphoid leukaemia | rs2651838 | T | A | 20.5 |  |
| Lymphoid leukaemia | rs4935666 | T | G | 21.8 |  |
| Lymphoid leukaemia | rs58192820 | G | A | 24.7 |  |
| Lymphoid leukaemia | rs59261129 | T | C | 19.6 |  |
| Lymphoid leukaemia | rs61915331 | T | C | 21.9 |  |
| Lymphoid leukaemia | rs62300272 | G | A | 20.1 |  |
| Lymphoid leukaemia | rs694519 | C | T | 20.4 |  |
| Lymphoid leukaemia | rs72643898 | T | C | 19.6 |  |
| Lymphoid leukaemia | rs73235929 | A | G | 21.9 |  |
| Lymphoid leukaemia | rs735665 | A | G | 26.2 |  |
| Lymphoid leukaemia | rs74860295 | C | T | 20.2 |  |
| Lymphoid leukaemia | rs763477 | C | A | 27.0 |  |
| Lymphoid leukaemia | rs78130692 | C | T | 20.1 |  |
| Lymphoid leukaemia | rs927096 | T | C | 20.0 |  |
| Lymphoid leukaemia | rs9866116 | G | A | 21.3 |  |
| Lymphoid leukaemia | rs9867613 | A | C | 20.8 |  |
| Lymphoid leukaemia | rs9930260 | A | G | 19.6 |  |
| Multiple myeloma | rs10078442 | C | T | 19.6 |  |
| Multiple myeloma | rs112575691 | T | C | 19.9 |  |
| Multiple myeloma | rs114121233 | C | T | 20.5 |  |
| Multiple myeloma | rs115253040 | T | C | 23.1 |  |
| Multiple myeloma | rs12162384 | A | C | 26.0 |  |
| Multiple myeloma | rs145294585 | C | T | 22.3 |  |
| Multiple myeloma | rs17704330 | T | G | 19.7 |  |
| Multiple myeloma | rs189058806 | T | A | 19.6 |  |
| Multiple myeloma | rs2578125 | T | C | 22.6 |  |
| Multiple myeloma | rs4877586 | A | G | 20.1 |  |
| Multiple myeloma | rs739794 | A | G | 19.9 |  |
| Multiple myeloma | rs7441672 | G | A | 22.4 |  |
| Multiple myeloma | rs7577599 | C | T | 20.5 |  |
| Multiple myeloma | rs76450314 | C | T | 22.4 |  |
| Multiple myeloma | rs79365144 | C | T | 19.9 |  |
| Non-Hodgkin lymphoma | rs111579211 | C | T | 19.7 |  |
| Non-Hodgkin lymphoma | rs112479596 | A | C | 23.9 |  |
| Non-Hodgkin lymphoma | rs113600650 | G | C | 22.4 |  |
| Non-Hodgkin lymphoma | rs114221742 | G | C | 23.0 |  |
| Non-Hodgkin lymphoma | rs1342494 | T | A | 26.3 |  |
| Non-Hodgkin lymphoma | rs145253828 | C | T | 21.3 |  |
| Non-Hodgkin lymphoma | rs145813058 | C | T | 21.4 |  |
| Non-Hodgkin lymphoma | rs146427908 | C | T | 24.6 |  |
| Non-Hodgkin lymphoma | rs188881087 | T | C | 19.7 |  |
| Non-Hodgkin lymphoma | rs2068813 | G | A | 21.1 |  |
| Non-Hodgkin lymphoma | rs2097442 | G | A | 80.4 |  |
| Non-Hodgkin lymphoma | rs3130388 | G | T | 21.0 |  |
| Non-Hodgkin lymphoma | rs34269949 | A | C | 26.4 |  |
| Non-Hodgkin lymphoma | rs34648969 | G | C | 21.0 |  |
| Non-Hodgkin lymphoma | rs3766377 | A | G | 21.8 |  |
| Non-Hodgkin lymphoma | rs6553961 | T | C | 23.7 |  |
| Non-Hodgkin lymphoma | rs77943404 | G | A | 24.9 |  |
| Non-Hodgkin lymphoma | rs78217738 | T | C | 21.3 |  |
| Non-Hodgkin lymphoma | rs80183370 | C | A | 22.5 |  |
| Non-Hodgkin lymphoma | rs9450293 | A | G | 19.9 |  |
| Non-Hodgkin lymphoma | rs9962462 | T | A | 19.8 |  |
| Pancreatic cancer | rs138159729 | G | A | 20.1 |  |
| Pancreatic cancer | rs138585571 | C | T | 26.5 |  |
| Pancreatic cancer | rs140244438 | G | A | 20.7 |  |
| Pancreatic cancer | rs140962918 | C | T | 23.8 |  |
| Pancreatic cancer | rs141264642 | G | A | 23.0 |  |
| Pancreatic cancer | rs146062430 | C | T | 20.9 |  |
| Pancreatic cancer | rs146702575 | G | T | 20.0 |  |
| Pancreatic cancer | rs148512905 | A | C | 27.9 |  |
| Pancreatic cancer | rs151085039 | C | T | 22.4 |  |
| Pancreatic cancer | rs17616130 | A | G | 22.0 |  |
| Pancreatic cancer | rs1884228 | T | C | 20.4 |  |
| Pancreatic cancer | rs2735948 | A | G | 31.7 |  |
| Pancreatic cancer | rs6032129 | C | G | 20.5 |  |
| Pancreatic cancer | rs635634 | C | T | 22.2 |  |
| Pancreatic cancer | rs71411601 | G | C | 27.1 |  |
| Pancreatic cancer | rs72816351 | C | G | 19.7 |  |
| Pancreatic cancer | rs76116565 | A | T | 20.4 |  |
| Pancreatic cancer | rs9573166 | G | A | 25.5 |  |
| Abbreviation: IVs: instrumental variables; EA: effect allele;  OA: other allele; SNP: single nucleotide polymorphism. | | | | | |

**Supplementary Table 3**. MR analysis results.

| Type of cancer | Method | Number of SNPs | Beta | SE | P |
| --- | --- | --- | --- | --- | --- |
| Overall cancer | IVW | 8 | -0.0386 | 0.0473 | 0.41 |
| Overall cancer | MR-Egger | 8 | 0.0028 | 0.0768 | 0.97 |
| Overall cancer | WM | 8 | 0.0358 | 0.0487 | 0.46 |
| Bladder cancer | IVW | 28 | -0.0006 | 0.0105 | 0.96 |
| Bladder cancer | MR-Egger | 28 | 0.0538 | 0.0318 | 0.10 |
| Bladder cancer | WM | 28 | 0.0004 | 0.0129 | 0.97 |
| Brain cancer | IVW | 20 | 0.0052 | 0.0039 | 0.18 |
| Brain cancer | MR-Egger | 20 | 0.0087 | 0.0058 | 0.15 |
| Brain cancer | WM | 20 | 0.0056 | 0.0053 | 0.29 |
| Esophagus/ stomach cancer | IVW | 25 | 0.0023 | 0.0056 | 0.68 |
| Esophagus/ stomach cancer | MR-Egger | 25 | 0.0069 | 0.0090 | 0.45 |
| Esophagus/ stomach cancer | WM | 25 | -0.0015 | 0.0079 | 0.85 |
| Lung cancer | IVW | 5 | -0.0167 | 0.0290 | 0.56 |
| Lung cancer | MR-Egger | 5 | -0.0372 | 0.1142 | 0.77 |
| Lung cancer | WM | 5 | -0.0284 | 0.0195 | 0.14 |
| Lymphoid leukaemia | IVW | 28 | 0.0075 | 0.0035 | 0.03 |
| Lymphoid leukaemia | MR-Egger | 28 | 0.0054 | 0.0052 | 0.31 |
| Lymphoid leukaemia | WM | 28 | 0.0054 | 0.0047 | 0.25 |
| Multiple myeloma | IVW | 15 | -0.0084 | 0.0049 | 0.09 |
| Multiple myeloma | MR-Egger | 15 | -0.0009 | 0.0077 | 0.91 |
| Multiple myeloma | WM | 15 | -0.0087 | 0.0067 | 0.20 |
| Non-Hodgkin lymphoma | IVW | 21 | 0.0174 | 0.0103 | 0.09 |
| Non-Hodgkin lymphoma | MR-Egger | 21 | -0.0024 | 0.0255 | 0.92 |
| Non-Hodgkin lymphoma | WM | 21 | 0.0172 | 0.0141 | 0.22 |
| Pancreatic cancer | IVW | 18 | 0.0048 | 0.0118 | 0.68 |
| Pancreatic cancer | MR-Egger | 18 | -0.0457 | 0.0209 | 0.04 |
| Pancreatic cancer | WM | 18 | 0.0033 | 0.0101 | 0.75 |
| Abbreviation: IVW: inverse-variance weighted method; MR: Mendelian randomization; WM: weighted median method; SNP: single nucleotide polymorphism; SE: standard error. | | | | | |

**Supplementary Table** **4**. Heterogeneity test results.

| Type of cancer | Q | Q_df | Q_P |
| --- | --- | --- | --- |
| Overall cancer | 13.96 | 7 | 5.19E-02 |
| Bladder cancer | 42.69 | 27 | 2.81E-02 |
| Brain cancer | 24.10 | 19 | 1.92E-01 |
| Esophagus/ stomach cancer | 27.69 | 24 | 2.73E-01 |
| Lung cancer | 13.96 | 4 | 7.41E-03 |
| Lymphoid leukaemia | 32.38 | 27 | 2.18E-01 |
| Multiple myeloma | 11.71 | 14 | 6.30E-01 |
| Non-Hodgkin lymphoma | 14.33 | 20 | 8.13E-01 |
| Pancreatic cancer | 55.50 | 17 | 5.69E-06 |

**Supplementary Table 5**. MR-Egger pleiotropy test results.

| Type of cancer | Egger_intercept | SE | P |
| --- | --- | --- | --- |
| Overall cancer | -0.0062 | 0.0089 | 0.51 |
| Bladder cancer | -0.0117 | 0.0065 | 0.08 |
| Brain cancer | -0.0031 | 0.0037 | 0.41 |
| Esophagus/ stomach cancer | -0.0029 | 0.0044 | 0.52 |
| Lung cancer | 0.0055 | 0.0294 | 0.86 |
| Lymphoid leukaemia | 0.0017 | 0.0030 | 0.58 |
| Multiple myeloma | -0.0052 | 0.0040 | 0.22 |
| Non-Hodgkin lymphoma | 0.0047 | 0.0055 | 0.40 |
| Pancreatic cancer | 0.0294 | 0.0107 | 0.01 |
| Abbreviation: MR: Mendelian randomization; SE: standard error. | | | |

**Supplementary Table 6**. MR-PRESSO test results.

| Type of cancer | Main.MR.results.MR.Analysis | Main.MR.results.Causal.Estimate | Main.MR.results.Sd | Main.MR.results.P.value |
| --- | --- | --- | --- | --- |
| Bladder cancer | Raw | -0.0006 | 0.0105 | 0.96 |
| Bladder cancer | Outlier-corrected | -0.0074 | 0.0098 | 0.46 |
| Pancreatic cancer | Raw | 0.0048 | 0.0118 | 0.69 |
| Pancreatic cancer | Outlier-corrected | -0.0052 | 0.0072 | 0.48 |
| Abbreviation: MR: Mendelian randomization. | | | | |
